# Serum YKL-40 in coronary heart disease: linkage with inflammatory cytokines, artery stenosis, and optimal cut-off value for estimating major adverse cardiovascular events

**DOI:** 10.3389/fcvm.2023.1242339

**Published:** 2023-10-31

**Authors:** Mowei Song, Guofu Zhang, Hang Shi, Erjun Zhu, Li Deng, Hongtao Shen

**Affiliations:** ^1^ Department of Cardiology, The First Affiliated Hospital, Harbin Medical University, Harbin, China; ^2^ Department of Cardiovascular Surgery, The First Affiliated Hospital, Harbin Medical University, Harbin, China; ^3^ Department of Cardiovascular Surgery, The Fourth Affiliated Hospital of Harbin Medical University, Harbin, China; ^4^ Department of Extracorporeal Life Support, The People’s Hospital of Gaozhou, Gaozhou, China; ^5^ Department of Orthopedic Surgery, The First Affiliated Hospital, Harbin Medical University, Harbin, China

**Keywords:** coronary heart disease, chitinase-3-like protein 1, disease features, inflammatory cytokines, major adverse cardiovascular events

## Abstract

**Objective:**

YKL-40, previously known as chitinase-3-like protein 1 (CHI3L1), is an inflammation-related glycoprotein that promotes atherosclerosis, but its application and optimal cut-off value as a prognostic biomarker in coronary heart disease (CHD) require more clinical evidence. Thus, this prospective study aimed to evaluate the linkage of serum YKL-40 with disease features, inflammatory cytokines, and major adverse cardiovascular events (MACEs) in CHD patients.

**Methods:**

A total of 410 CHD patients were enrolled for serum YKL-40 determination via enzyme-linked immunosorbent assay. Meanwhile, serum YKL-40 levels in 100 healthy controls (HCs) were also quantified.

**Results:**

YKL-40 level was higher in CHD patients compared with that in HCs (*P *< 0.001). YKL-40 was positively linked with hyperlipidemia (*P *= 0.014), diabetes mellitus (*P *= 0.001), fasting blood glucose (*P *= 0.045), C-reactive protein (*P *< 0.001), the Gensini score (*P *< 0.001), and stenosis degree (graded by the Gensini score) (*P *< 0.001) in CHD patients. In addition, an elevated YKL-40 level was associated with increased levels of tumor necrosis factor alpha (*P *= 0.001), interleukin (IL)-1β (*P *= 0.001), IL-6 (*P *< 0.001), and IL-17A (*P *= 0.002) in CHD patients. The 1-/2-/3-year cumulative MACE rates of CHD patients were 5.5%, 14.4%, and 25.0%, respectively. Regarding the prognostic capability, YKL-40 ≥100 ng/ml (the median cut-off value) (*P *= 0.003) and YKL-40 ≥150 ng/ml (the third interquartile cut-off value) (*P *= 0.021) reflected an elevated accumulating MACE rate, whereas accumulating MACE was not different between CHD patients with YKL-40 ≥80 and <80 ng/ml (the first interquartile cut-off value) (*P *= 0.083).

**Conclusion:**

Serum YKL-40 is positively linked with inflammatory cytokines and the Gensini score, whose high expression cut-off by 100 and 150 ng/ml estimates a higher MACE risk in CHD patients.

## 1. Introduction

Coronary heart disease (CHD), which consists of stable angina and acute coronary syndromes, is a series of life-threatening diseases that account for approximately 7 million deaths globally each year (1, 2). CHD is caused by the interaction of complex pathogenic factors, such as cholesterol-rich apolipoprotein B (ApoB) accumulation within the arterial intima, chronic inflammation, vascular endothelial dysfunction, and other factors contributing to atherogenesis and CHD (3–5). Despite appropriate application of lifestyle, pharmacological, or surgical interventions, the major cardiovascular outcomes of CHD remain unsatisfactory (6, 7). Thus, continued efforts in seeking biomarkers that estimate the clinical prognosis of CHD patients are still necessary.

YKL-40, previously named chitinase-3-like protein 1 (CHI3L1), is an inflammation-related glycoprotein that belongs to the glycoside hydrolase family and is involved in CHD progression as noted in several studies (8–10). A previous study showed that YKL-40 increases the lesion area of atherosclerotic plaques in an apolipoprotein E-deficient (ApoE^−/−^) mouse model (9). Another study revealed that YKL-40 exacerbates atherosclerosis by inducing endothelial cell inflammation and activation of vascular smooth muscle cells (10).

Some clinical studies have identified the ability of YKL-40 to identify the disease risk and severity of CHD (11–14). In addition, the prognostic value of YKL-40 for CHD patients cannot be ignored (15–17). For example, one study determined YKL-40 in patients with ST-segment elevation myocardial infarction (STEMI) and found that it is positively linked with in-hospital major adverse cardiovascular events (MACEs) in these patients (16). Another study revealed the predictive value of YKL-40 for mortality in stable CHD patients (17). Nonetheless, these studies focused on either stable CHD or STEMI (15–17), and the optimal cut-off value of YKL-40 requires more clinical evidence.

Hence, this prospective study quantified serum YKL-40 in 410 CHD patients to observe the linkage of serum YKL-40 with disease features, inflammatory cytokines, and MACE in these patients.

## 2. Methods

### 2.1. Subjects

A total of 410 patients with CHD diagnosed by coronary angiography between January 2019 and October 2022 were consecutively enrolled in this research. The patients were included if they were (i) diagnosed with CHD via coronary angiography; (ii) aged more than 18 years old; and (iii) about to and able to participate. The patients were excluded if they (i) had malignant diseases, (ii) had pronounced infections, or (iii) were pregnant or lactating. In addition, 100 people who recently underwent physical examination in our hospital were enrolled as healthy controls (HCs). The inclusion criteria of HCs were as follows: (i) those without abnormalities in the physical examination; (ii) those who were age-matched and gender-matched with the CHD patients and aged from 45 to 79 years old with a male-to-female ratio of 7:3; and (iii) those who were ready to cooperate with this research. HCs who have histories of drug abuse, are pregnant, or are lactating were excluded. The Ethics Committee of the First Affiliated Hospital, Harbin Medical University, supported this research (approval No. 2018098). Informed consent was obtained from each subject.

### 2.2. Data collection

Demographics, medical history, biochemical indexes, and disease characteristics were collected from the CHD patients. In addition, the Gensini score was collected by coronary arteriography to assess the luminal stenosis, which was the sum of all lesion scores, and was further graded into mild (<32), moderate (32–56), and severe (>56). Each lesion score was completed by multiplying the stenosis score and the lesion site score (18).

### 2.3. Sample collection and determination

Peripheral blood (PB) samples were gathered from the CHD patients at enrollment during the acute phase; meanwhile, PB samples of HCs were also obtained at enrollment. Thereafter, PB samples were isolated for serum analysis to detect YKL-40. Serum YKL-40 was detected via enzyme-linked immunosorbent assay (ELISA) using a commercial kit (Cat. No. DY2599, R&D, USA). For the majority of the CHD patients (*n* = 354), inflammatory cytokines including interleukin (IL)-1β, tumor necrosis factor alpha (TNF-α), IL-6, and IL-17A were also measured by ELISA. The ELISA kits used were obtained from the R&D Systems (USA), and the catalog numbers of the kits were DTA00D, HSLB00D, D6050, and QK317. All tests were performed in triplicate and were strictly conducted in accordance with the instructions of the kit.

### 2.4. Follow-up and evaluation

The CHD patients underwent routine follow-ups (median, 15.5 months; range, 1.1–43.7 months). During follow-ups (last follow-up date: December 2022), MACEs were recorded and defined similarly in previous research (19), including cardiovascular death, myocardial infarction, unplanned coronary revascularization, and hospital admission for cardiovascular causes. In addition, the cumulative MACE risk was calculated for evaluation.

### 2.5. Cut-off values

For the CHD patients, the median value of YKL-40 was approximately 100 ng/ml, the first interquartile (Q1) value of YKL-40 was approximately 80 ng/ml, and the third interquartile (Q3) value of YKL-40 was approximately 150 ng/ml. For prognosis analysis, YKL-40 was classified into different levels with the following cut-off values: (1) median cut-off value, ≥100 and <100 ng/ml levels; (2) Q1 cut-off value, ≥80 and <80 ng/ml levels; and (3) Q3 cut-off value, ≥150 and <150 ng/ml levels.

### 2.6. Statistics

SPSS 26.0 (IBM, USA) and GraphPad Prism 7.0.1 (GraphPad Software, USA) were utilized for data processing and figure plotting, respectively. The Wilcoxon rank-sum test or Kruskal–Wallis test was used to estimate the difference between the two or among multiple groups as appropriate. Student’s *t*-test and *χ*^2^ test were used to compare age and gender between the CHD patients and HCs, respectively. The ability of YKL-40 to distinguish between CHD patients and HCs was recognized by a plotted receiver operating characteristic (ROC) curve. In addition, the ability of YKL-40 to predict 1-year, 2-year, and 3-year MACE risks was also evaluated by ROC curves. Correlation analysis was conducted using the Spearman test. Kaplan–Meier curves were performed to show the accumulating MACE rates, in which the log-rank test was used to compare the accumulating MACE rates between patients with different YKL-40 levels. Univariate and stepwise forward multivariate Cox regression analyses were conducted to identify the influence factors of MACE. *P* < 0.050 indicated statistical significance.

## 3. Results

### 3.1. Clinical features of CHD patients and HCs

There were 122 (29.8%) females and 288 (70.2%) males among the 410 CHD patients whose mean age was 62.9 ± 9.9 years. The median [interquartile range (IQR)] Gensini score was 32.3 (17.0–50.1). In 104 CHD patients with diabetes mellitus (DM), the body mass index (BMI) of 61 (58.7%) and 23 (22.1%) patients was ≥24 kg/m^2^ and ≥28 kg/m^2^, respectively. In addition, 199 (48.5%), 124 (30.2%), and 87 (21.3%) patients were classified as having mild, moderate, and severe stenosis degrees, respectively, based on the Gensini score. In addition, 410 (100.0%), 289 (70.5%), 116 (28.3%), 177 (43.2%), and 157 (38.3%) CHD patients received antiplatelet therapy, β-blocker, calcium channel blockers, statin or other lipid-lowering therapy, and angiotensin-converting enzyme inhibitor (ACEI) or angiotensin II receptor blocker (ARB), respectively. Table 1 provides detailed information on the CHD patients.

**Table 1:** Clinical characteristics of CHD patients.

Items	CHD patients (*N* = 410)
Demographics	
Age (years), mean ± SD	62.9 ± 9.9
Gender, No. (%)	
Female	122 (29.8)
Male	288 (70.2)
BMI (kg/m^2^), mean ± SD	25.5 ± 3.1
History of smoke, No. (%)	
Never	207 (50.5)
Former	120 (29.3)
Current	83 (20.2)
Medical history	
History of hypertension, No. (%)	
No	122 (29.8)
Yes	288 (70.2)
History of hyperlipidemia, No. (%)	
No	233 (56.8)
Yes	177 (43.2)
History of DM, No. (%)	
No	306 (74.6)
Yes	104 (25.4)
History of CKD, No. (%)	
No	339 (82.7)
Yes	71 (17.3)
Biochemical indexes	
FBG (mmol/L), median (IQR)	6.0 (5.2–7.0)
Scr (μmol/L), median (IQR)	84.7 (75.3–93.3)
SUA (μmol/L), median (IQR)	353.0 (305.0–410.3)
TG (mmol/L), median (IQR)	1.8 (1.1–2.5)
TC (mmol/L), median (IQR)	4.9 (4.2–5.7)
LDL-C (mmol/L), median (IQR)	3.5 (2.9–4.3)
HDL-C (mmol/L), median (IQR)	0.9 (0.8–1.1)
CRP (mg/L), median (IQR)	5.6 (3.9–8.1)
Disease characteristics	
Gensini score, median (IQR)	32.3 (17.0–50.1)
Stenosis degree	
Mild (Gensini score <32), No. (%)	199 (48.5)
Moderate (Gensini score 32–56), No. (%)	124 (30.2)
Severe (Gensini score >56), No. (%)	87 (21.3)
Inflammatory cytokines	
TNF-α (pg/ml), median (IQR)	
IL-1β (pg/ml), median (IQR)	1.0 (0.6–1.5)
IL-6 (pg/ml), median (IQR)	16.9 (12.6–26.7)
IL-17A (pg/ml), median (IQR)	58.6 (43.7–90.9)
Current treatment information	
Antiplatelet therapy	410 (100.0)
β-Blocker	289 (70.5)
Calcium channel blockers	116 (28.3)
Statin or other lipid-lowering therapy	177 (43.2)
ACEI or ARB	157 (38.3)

SD, standard deviation; Scr, serum creatinine; TG, triglyceride; TC, total cholesterol; HDL-C, high-density lipoprotein cholesterol.

In addition, there were 30 (30.0%) females and 70 (70.0%) males in HCs, with a mean age of 61.6 ± 8.2 years. Meanwhile, age (*P *= 0.222) and gender (*P *= 0.962) had no significant difference between the CHD patients and HCs (Supplementary Table S1).

### 3.2. Comparison of serum YKL-40 levels between CHD patients and HCs

**Figure 1:**
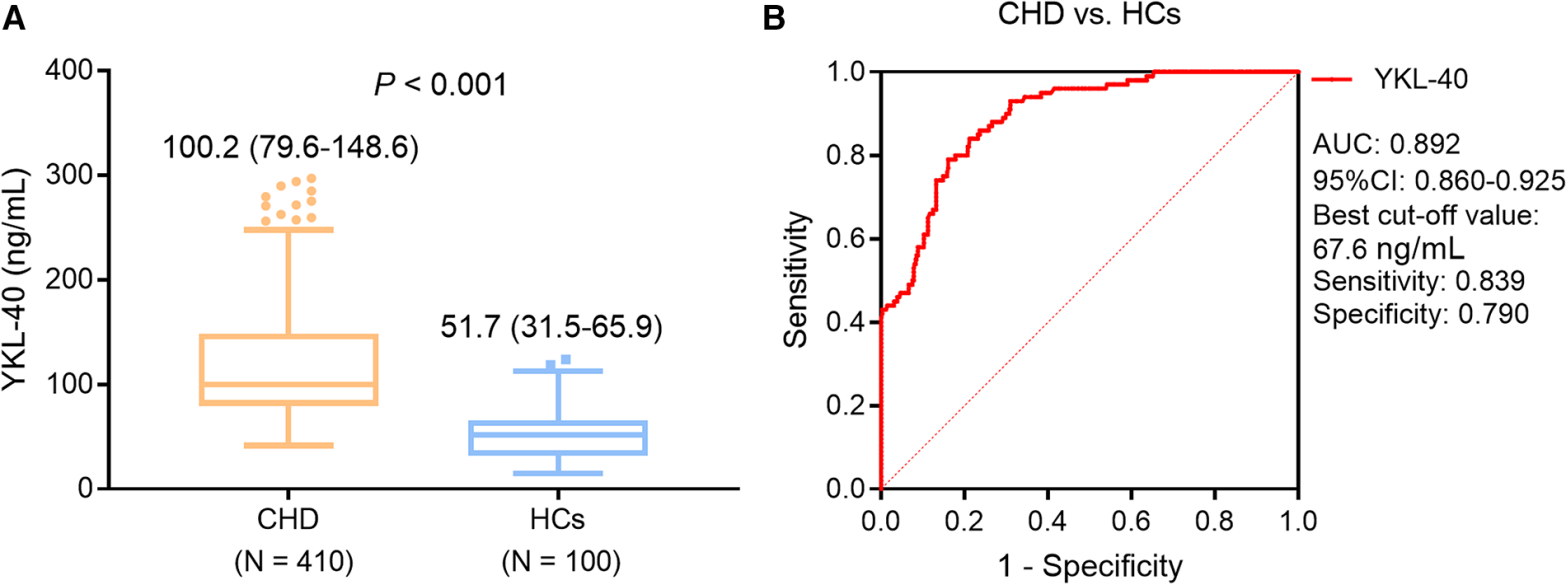
Serum YKL-40 level was higher in CHD patients compared with that in HCs. Comparison of serum YKL-40 between CHD patients and HCs. (**A**) ROC curve for the ability of serum YKL-40 to differentiate CHD patients from HCs (**B**).

Serum YKL-40 level was higher in the CHD patients compared with that in HCs [median (IQR): 100.2 (79.6–148.6) ng/ml vs. 51.7 (31.5–65.9) ng/ml, *P *< 0.001] (Figure 1A). Meanwhile, serum YKL-40 possessed a pleasing ability to distinguish CHD patients from HCs [area under the curve (AUC): 0.892, 95% confidence interval (CI): 0.860–0.925] (Figure 1B).

### 3.3. Linkage of serum YKL-40 with clinical characteristics in CHD patients

**Figure 2:**
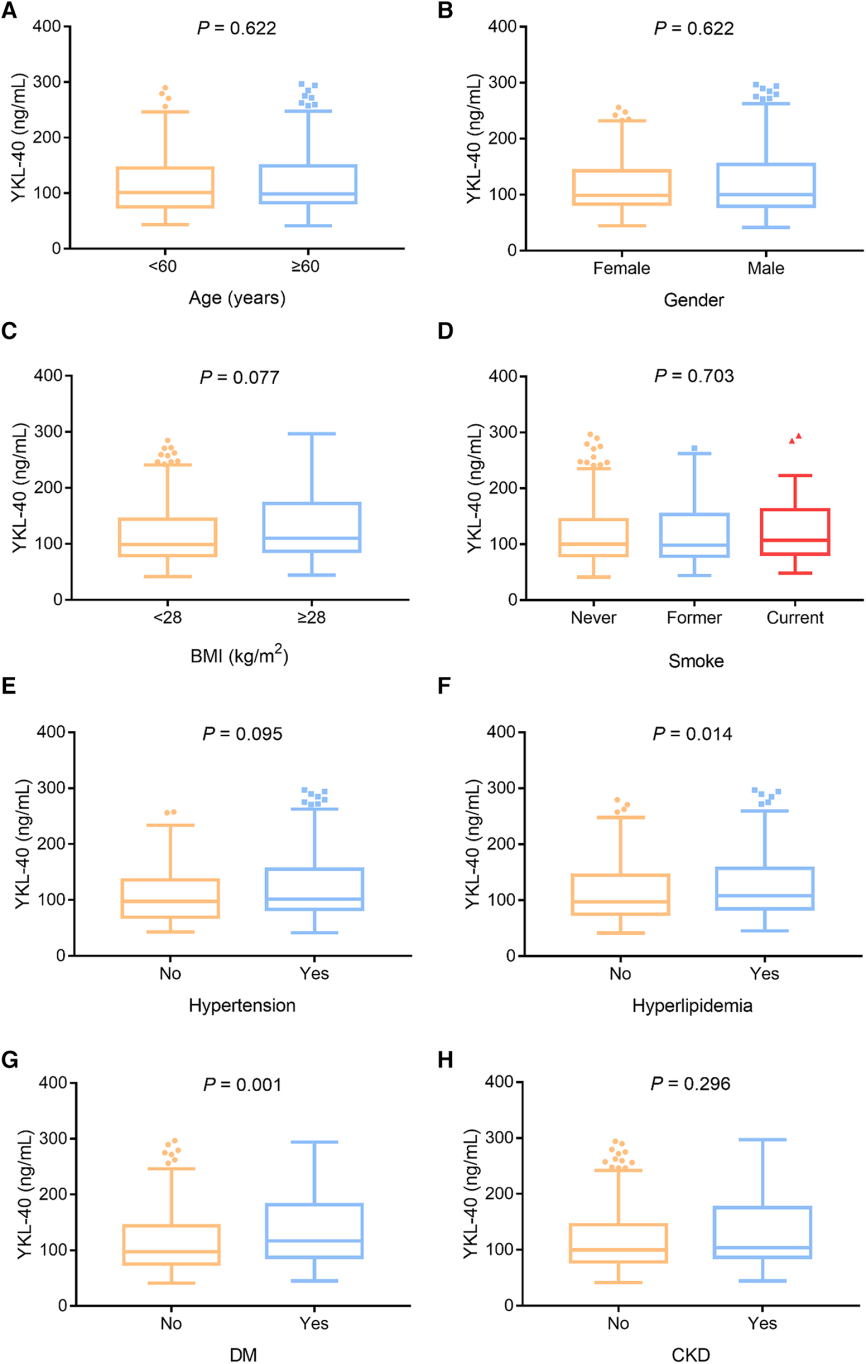
Serum YKL-40 was positively linked with hyperlipidemia and DM in CHD patients. Correlation of serum YKL-40 with age (**A**), sex (**B**), BMI (**C**), smoking (**D**), hypertension (**E**), hyperlipidemia (**F**), DM (**G**), and CKD (**H**) in CHD patients.

Serum YKL-40 was positively linked with hyperlipidemia (*P *= 0.014) and DM (*P *= 0.001) but not with age (*P *= 0.622), sex (*P *= 0.622), BMI (*P *= 0.077), smoking (*P *= 0.703), hypertension (*P *= 0.095), or chronic kidney disease (CKD) (*P *= 0.296) in CHD patients (Figures 2A–H). In addition, serum YKL-40 was positively correlated with fasting blood glucose (FBG) (*P *= 0.045) and C-reactive protein (CRP) (*P *< 0.001) but not with the other biochemical indexes in CHD patients (all *P *> 0.050) (Table 2). Moreover, serum YKL-40 was not related to β-blocker (*P *= 0.273), calcium channel blockers (*P *= 0.296), statin or other lipid-lowering therapy (*P *= 0.061), or ACEI or ARB (*P *= 0.186) in CHD patients (Supplementary Table S2).

**Table 2:** Correlation of YKL-40 with biochemical indexes in CHD patients.

Items	*r*-value	*P*-value
FBG (mmol/L)	0.099	0.045
Scr (μmol/L)	0.056	0.262
SUA (μmol/L)	0.072	0.145
TG (mmol/L)	0.065	0.186
TC (mmol/L)	0.090	0.069
LDL-C (mmol/L)	0.093	0.063
HDL-C (mmol/L)	−0.028	0.566
CRP (mg/L)	0.227	<0.001

Scr, serum creatinine; TG, triglyceride; TC, total cholesterol; HDL-C, high-density lipoprotein cholesterol.

The data were analyzed using Spearman's rank correlation test and displayed as *r*-value and *P*-value.

### 3.4. Linkage of serum YKL-40 with artery stenosis and inflammation in CHD patients

**Figure 3:**
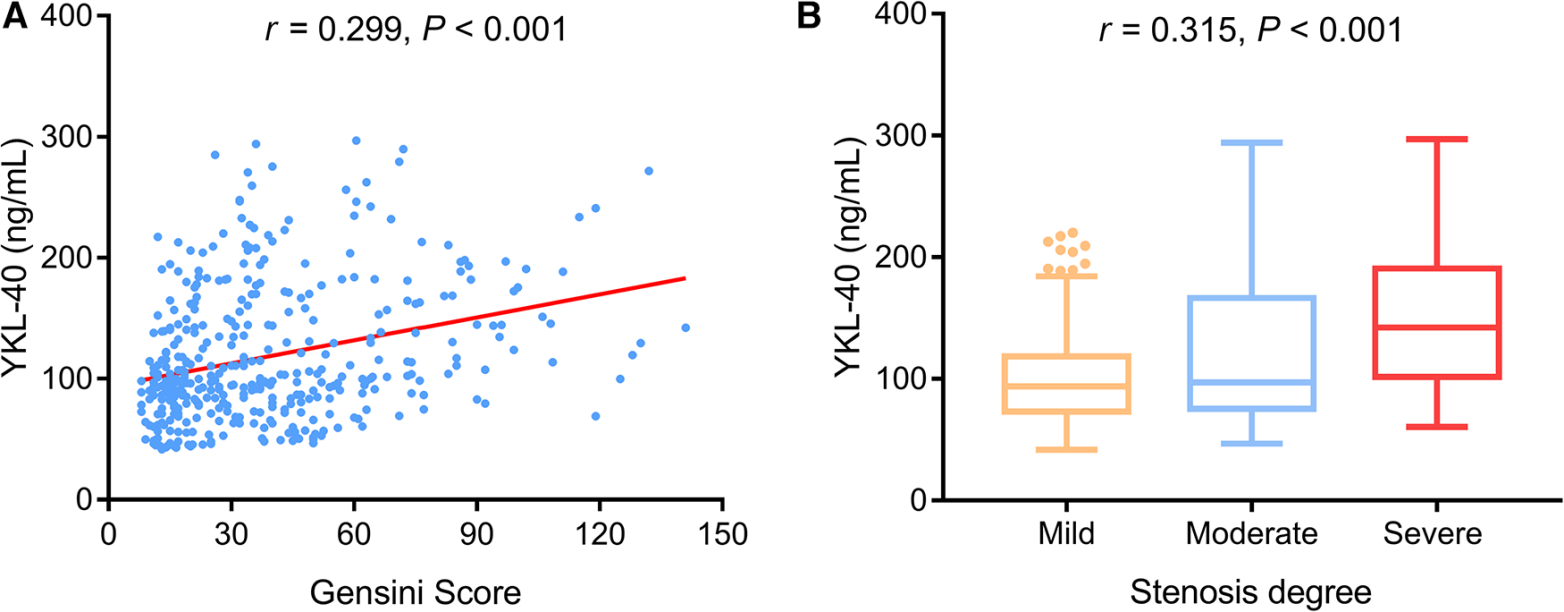
Serum YKL-40 was positively related to the Gensini score and stenosis degree in CHD patients. Association of serum YKL-40 with Gensini score (**A**) and stenosis degree (**B**) in CHD patients.

Serum YKL-40 was positively associated with the Gensini score (*P *< 0.001) (Figure 3A) and stenosis degree (*P *< 0.001) (Figure 3B) in CHD patients.

**Figure 4:**
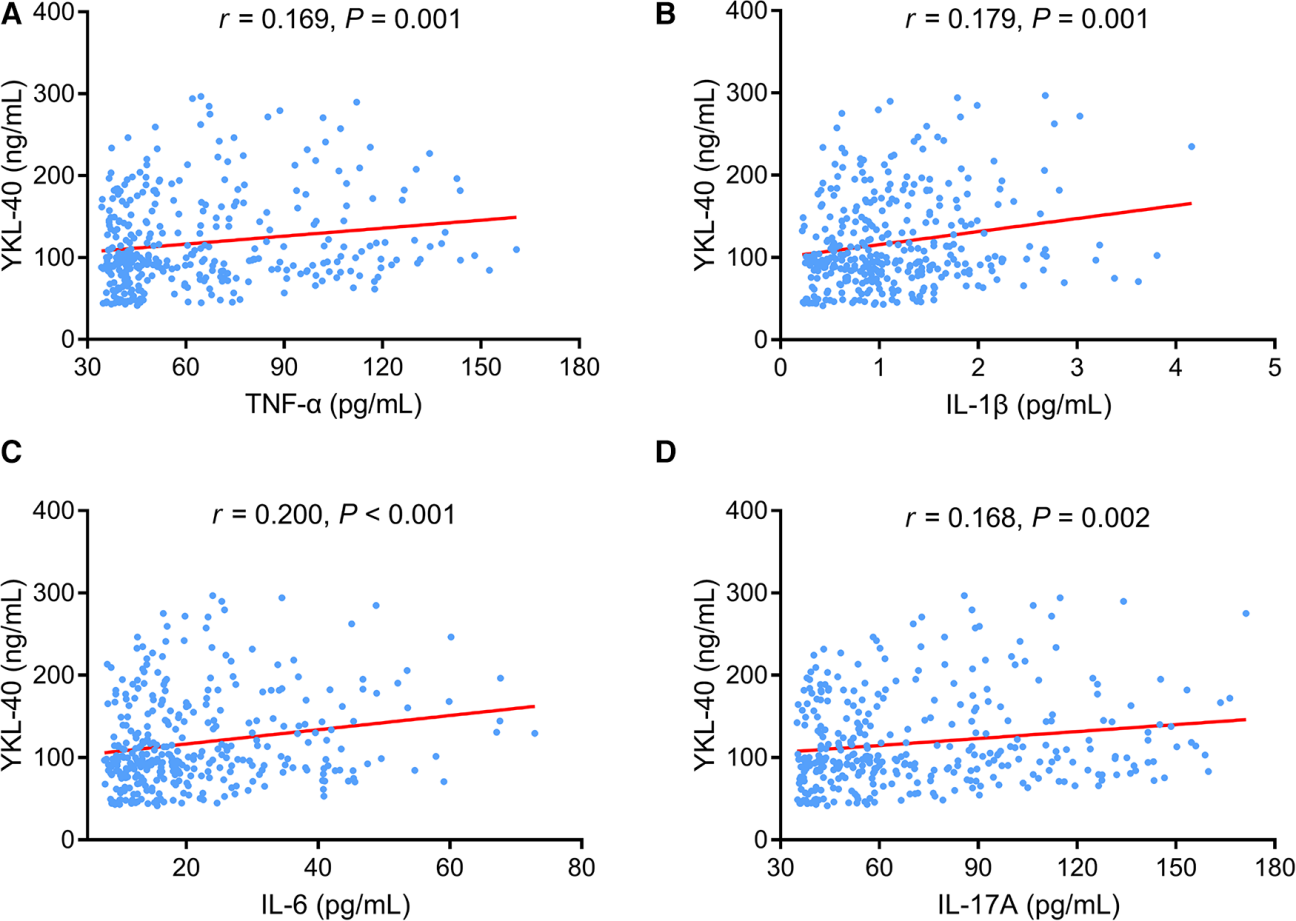
Serum YKL-40 positively correlated with TNF-α, (IL-1β, IL-6, and IL-17A in CHD patients. Linkage of serum YKL-40 with TNF-α (**A**), IL-1β (**B**), IL-6 (**C**), and IL-17A (**D**) in CHD patients.

With regard to the inflammatory cytokines, elevated serum YKL-40 level was linked with increased TNF-α (*P *= 0.001) (Figure 4A), IL-1β (*P *= 0.001) (Figure 4B), IL-6 (*P *< 0.001) (Figure 4C), and IL-17A (*P *= 0.002) (Figure 4D) in CHD patients.

### 3.5. Potency of serum YKL-40 for estimating MACE in CHD patients

**Figure 5:**
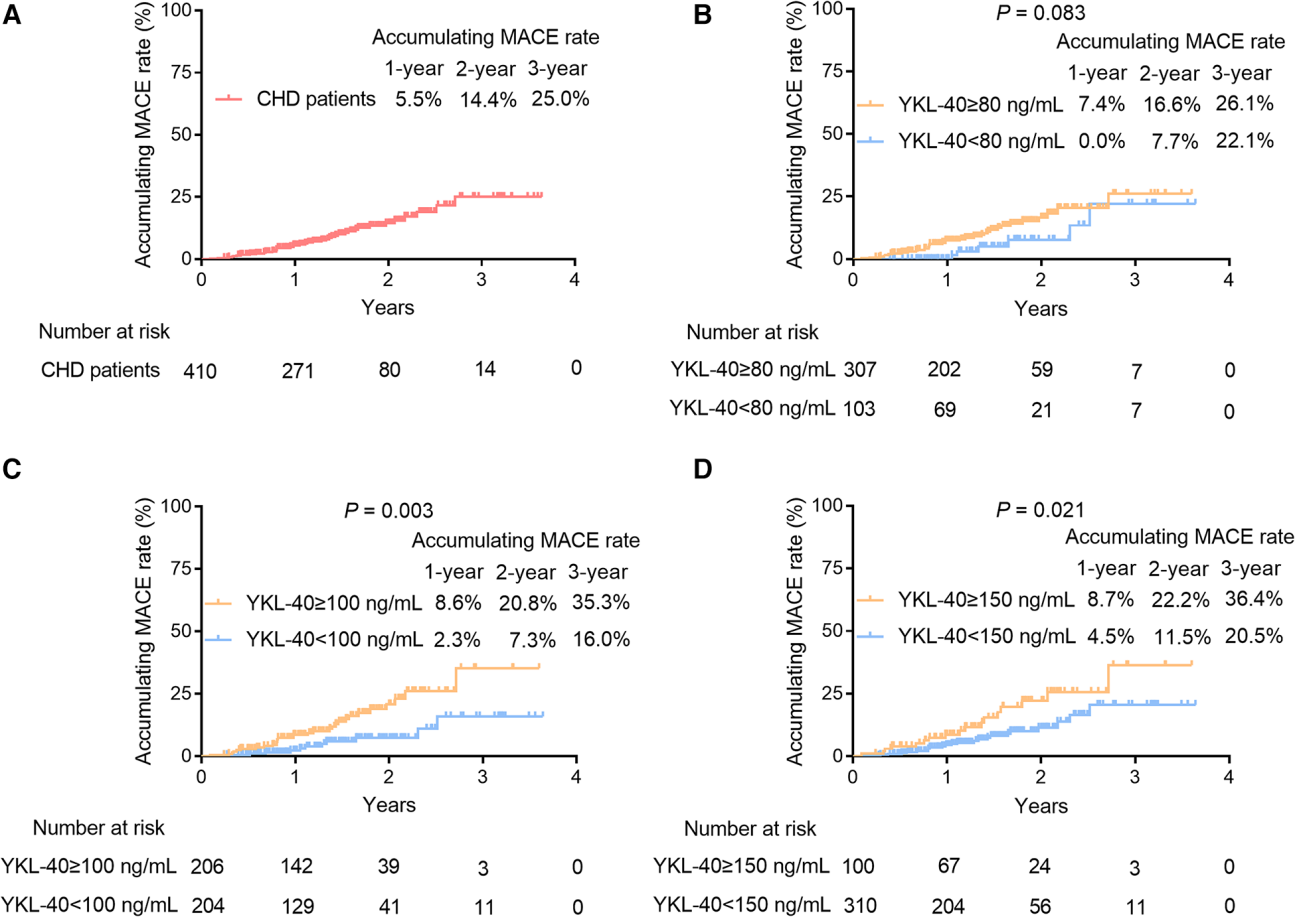
Serum YKL-40 ≥100 and ≥150 ng/ml were associated with increased MACE accumulation in CHD patients. The 1-/2-/3-year cumulative MACE rate in CHD patients (**A**) Prognostic value of serum YKL-40 cut-off values of 80 ng/ml (**B**), 100 ng/ml (**C**), and 150 ng/ml (**D**) in CHD patients.

Among the 41 CHD patients who had MACE, there were five patients with cardiovascular death, 11 patients with myocardial infarction, 17 patients with unplanned coronary revascularization, and eight patients with hospital admission for cardiovascular causes. The 1-/2-/3-year cumulative MACE rates of CHD patients were 5.5%, 14.4%, and 25.0%, respectively (Figure 5A). Moreover, serum YKL-40 was cut off by its Q1 (80 ng/ml), median (100 ng/ml), and Q3 (150 ng/ml) values in CHD patients to explore its prognostic value. Accumulating MACE rates did not vary between CHD patients with serum YKL-40 levels of ≥80 and <80 ng/ml (*P *= 0.083). In detail, the 1-/2-/3-year accumulating MACE rates were 7.4%, 16.6%, and 26.1%, respectively, in patients with a serum YKL-40 level of ≥80 ng/ml and 0.0%, 7.7%, and 22.1%, respectively, in patients with a serum YKL-40 level of <80 ng/ml (Figure 5B). In contrast, a serum YKL-40 level of ≥100 ng/ml was linked with elevated MACE in CHD patients (*P *= 0.003). The 1-/2-/3-year accumulating MACE rates were 8.6%, 20.8%, and 35.3%, respectively, in patients with a serum YKL-40 level of ≥100 ng/ml and 2.3%, 7.3%, and 16.0%, respectively, in patients with a serum YKL-40 level of <100 ng/ml (Figure 5C). In addition, a serum YKL-40 level of ≥150 ng/ml was correlated with increased MACE risk in CHD patients (*P *= 0.021). Specifically, the 1-, 2-, and 3-year cumulative MACE rates were 8.7%, 22.2%, and 36.4%, respectively, in patients with a serum YKL-40 level of ≥150 ng/ml and 4.5%, 11.5%, and 20.5%, respectively, in patients with a serum YKL-40 level of <150 ng/ml (Figure 5D). Furthermore, when YKL-40 was considered as a continuous variable, elevated YKL-40 level was correlated with increased MACE risk (hazard ratio: 1.007, 95% CI: 1.002–1.011, *P *= 0.006).

**Figure 6:**
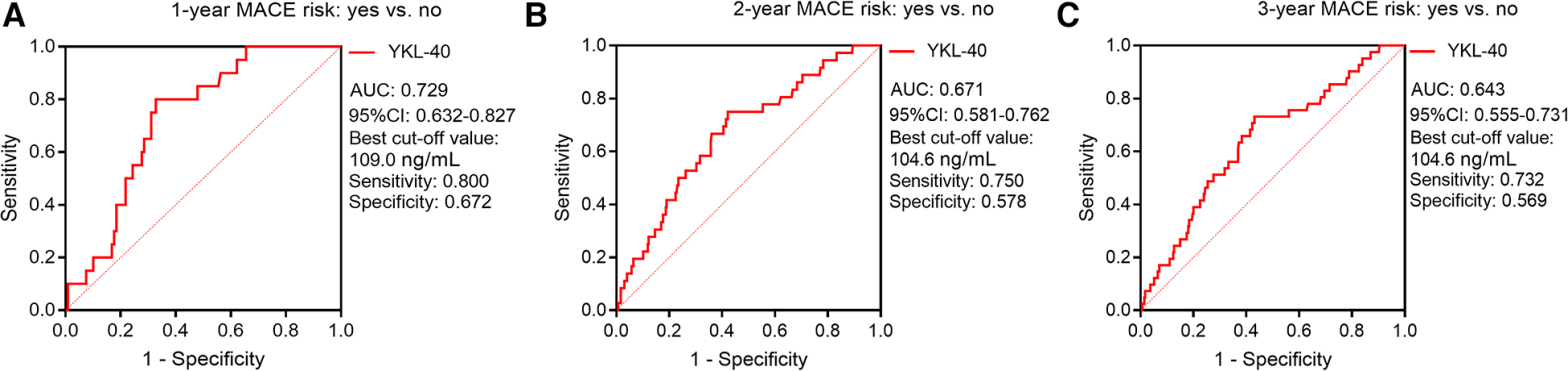
Serum YKL-40 possessed a good value for predicting 1-/2-/3-year MACE risks. ROC curves for the ability of serum YKL-40 to estimate 1-year (**A**), 2-year (**B**), and 3-year (**C**) MACE risks in CHD patients.

Furthermore, ROC curves showed that serum YKL-40 had a good value for predicting 1-year MACE risk (AUC, 0.729; best cut-off value, 109.0 ng/ml; sensitivity, 0.800; specificity, 0.672) (Figure 6A); meanwhile, serum YKL-40 disclosed a general capability for estimating 2-year (AUC, 0.671; best cut-off value, 104.6 ng/ml; sensitivity, 0.750; specificity, 0.578) (Figure 6B) and 3-year (AUC, 0.643; best cut-off value, 104.6 ng/ml; sensitivity, 0.732; specificity, 0.569) (Figure 6C) MACE risk in CHD patients.

### 3.6. Influencing factors for MACE in CHD patients

The univariate Cox regression analysis revealed that YKL-40 ≥100 ng/ml (*P *= 0.005) and YKL-40 ≥150 ng/ml (*P *= 0.024) were linked with increased MACE risk in CHD patients. However, the stepwise forward multivariate Cox regression analysis exhibited that YKL-40 was not an independent influence factor for MACE in CHD patients (*P *> 0.050). History of smoke (former or current vs. never) (*P *= 0.014), CRP ≥5 mg/L (*P *= 0.024), and higher Gensini score (*P *= 0.002) were independently associated with increased MACE risk, while serum uric acid (SUA) >350 μmol/L (*P *= 0.049) and low-density lipoprotein cholesterol (LDL-C) ≥2.60 mmol/L (*P *= 0.015) were related to declined MACE risk in CHD patients (Supplementary Table S3).

Moreover, the combination of YKL-40 ≥100 ng/ml, history of smoke, SUA >350 μmol/L, LDL-C ≥2.60 mmol/L, CRP ≥5 mg/L, and higher Gensini score disclosed a general value for estimating 3-year MACE risk in CHD patients (AUC, 0.620) (Supplementary Figure S1A), so did the combination of YKL-40 ≥50 ng/ml, history of smoke, SUA >350 μmol/L, LDL-C ≥2.60 mmol/L, CRP ≥5 mg/L, and higher Gensini score (AUC, 0.603) (Supplementary Figure S1B).

## 4. Discussion

The engagement of YKL-40 in several chronic diseases has been reported before (20, 21). A previous study reported that elevated YKL-40 is related to the presence and severity of metabolic syndrome (20). Another study suggested that YKL-40 is associated with all lipoprotein subclasses in patients with type I DM (21). CHD patients are often complicated with dyslipidemia and metabolic syndrome (22). The present study revealed that serum YKL-40 is positively related to hyperlipidemia, DM, and FBG in CHD patients. These results could be explained as follows: (1) YKL-40 increased the concentration of lipoprotein subclasses, which are crucial in the pathological process of hyperlipidemia (21, 23). Hence, serum YKL-40 was positively linked with hyperlipidemia in CHD patients. (2) YKL-40 suppressed insulin-mediated glucose metabolism by cross talk with intestinal fatty acid binding proteins (24, 25). Therefore, increased serum YKL-40 was linked with DM and increased FBG in CHD patients. These findings indicated that targeting YKL-40 might contribute to the reduction of the risk of complications (such as dyslipidemia and metabolic syndrome) in CHD patients.

Moreover, this study also showed that serum YKL-40 was positively linked with the Gensini score and stenosis degree in CHD patients, which could be explained by the fact that YKL-40 accelerated atherosclerotic plaque initiation and deterioration by inhibiting macrophage apoptosis, which aggravated luminal stenosis (9, 26, 27). As a result, serum YKL-40 was positively related to the Gensini score and stenosis degree in CHD patients.

YKL-40, secreted by locally activated macrophages and neutrophils, is a well-recognized inflammatory glycoprotein involved in both chronic and acute inflammation, and its positive correlation with many inflammatory cytokines, such as IL-6 and IL-18, has been observed previously (28–30). Also, it is exhibited that YKL-40 activates the nuclear transcription factor-kappa B (NF-κB) signaling pathway by promoting the NF-κB subunit nuclear translocation (31, 32). This study showed that serum YKL-40 was positively linked with TNF-α, IL-1β, IL-6, IL-17A, and CRP in CHD patients. The probable explanations were that (1) YKL-40 facilitated vascular inflammation, and thereafter, proinflammatory cytokines all surged in CHD patients (10, 33). As a result, serum YKL-40 was positively related to these inflammatory cytokines in CHD patients. (2) CRP is a systemic inflammatory marker secreted by hepatocytes in response to IL-6, and in this study, IL-6 was positively associated with serum YKL-40 in CHD patients (34). Consequently, a positive correlation was seen between serum YKL-40 and CRP in CHD patients.

The prognostic value of YKL-40 in cardiovascular diseases has been elucidated in some previous studies (35–37). For instance, one study found a positive linkage of YKL-40 with all-cause mortality in aortic stenosis patients (35). Similarly, another study showed that elevated YKL-40 is a risk factor for cardiovascular death in chronic CHD patients (36). The present study showed that increased serum YKL-40 was related to elevated MACE in CHD patients. The probable explanations are as follows: (1) Elevated YKL-40 was linked with aggravated inflammatory lesions and a more severe degree of artery stenosis, and the latter were considered risk factors for elevated MACE in CHD patients (16). (2) YKL-40 was a mediator of plaque vulnerability that increased the rupture risk of the fibrous cap, and then the MACE risk was elevated (38). Combining the above two reasons, serum YKL-40 was positively related to MACE risk in CHD patients. However, the stepwise forward multivariate Cox regression analysis exhibited that YKL-40 was not an independent influence factor for MACE in CHD patients, indicating that YKL-40 might exert its prognostic value with the interaction with lipid and inflammation.

Consistent with the previous studies (39, 40), this study set the cut-off value of YKL-40 by the median and interquartile values. Interestingly, serum YKL-40 cut-offs of 100 ng/ml (median) and 150 ng/ml (3/4 interquartile) had a good ability to estimate MACE in CHD patients, but when it was a cut-off of 80 ng/ml (1/4 interquartile), it lacked predictive value. The findings suggested that 100 ng/ml and 150 ng/ml serum YKL-40 levels appeared to be candidate prognostic biomarkers, and patients with YKL-40 levels of ≥100 and 150 ng/ml needed more close monitoring. Further verification was necessary.

There are some shortcomings in the current study. First, the follow-up period (median, 15.5 months) was too short to collect enough MACEs, which might result in underestimated MACE rate. Second, the measurement of serum YKL-40 in CHD patients was only conducted at enrollment during the acute phase, but its fluctuation during the CHD course was unknown, which might be an additional limitation in the current study. Third, because the comparison of serum YKL-40 between CHD patients and HCs was not the main objective of this study, the ratio of CHD patients and HCs was not balanced. Fourth, though YKL-40 disclosed good discriminating value between CHD patients and HCs, whether it was superior to a common blood test was unclear. Fifth, *in vitro* and *in vivo* experiments were not conducted in this study to investigate the underlying mechanism of YKL-40 in CHD. Sixth, this study lacked external validation in independent cohorts, which affected the generalization of the findings. Seventh, this study did not enroll disease controls, and confounders might exist. Eighth, according to the previous study (41), the YKL-40 level gradually decreased after exercise in CHD patients, but this issue was not explored in the current study.

Conclusively, increased serum YKL-40 level relates to elevated inflammatory cytokines and exacerbated artery stenosis, whose high expression cut-offs of 100 and 150 ng/ml reflect higher MACE risk in CHD patients. The findings indicate that serum YKL-40 may serve as a potential biomarker for identifying CHD patients with a high risk of unfavorable prognosis, which helps provide individualized management to these patients.

## Data Availability

The original contributions presented in the study are included in the article/**Supplementary Material**, further inquiries can be directed to the corresponding authors.

## Supplementary material

Supplementary Figure S1 Combination of serum YKL-40 and independent factors showed a general value for estimating 3-year MACE risks. The ROC curve for the ability of the combination of YKL-40 ≥100 ng/mL, smoke, SUA >350 μmol/L, LDL-C ≥2.60 mmol/L, CRP ≥5 mg/L, and higher Gensini score to predict 3-year MACE risk in CHD patients (**A**). The ROC curve for the ability of the combination of YKL-40 ≥150 ng/mL, smoke, SUA >350 μmol/L, LDL-C ≥2.60 mmol/L, CRP ≥5 mg/L, and higher Gensini score to predict 3-year MACE risk in CHD patients (**B**). Fix correct style.
