# Supplementary material for: Serum YKL-40 in coronary heart disease: linkage with inflammatory cytokines, artery stenosis, and optimal cut-off value for estimating major adverse cardiovascular events
Source: Front Cardiovasc Med. 2023 Oct 31;10:1242339. doi: 10.3389/fcvm.2023.1242339 (PMC10644235; doi:10.3389/fcvm.2023.1242339)
Supplement: Supplementary file 1 [file Table1.docx]

**Supplementary Table 1.** Comparison of age and gender between CHD patients and HCs at baseline.

| Items | HCs  (N = 100) | CHD patients  (N = 410) | *P* value |
| --- | --- | --- | --- |
| Age (years), mean±SD | 61.6±8.2 | 62.9±9.9 | 0.222 |
| Gender, No. (%) |  |  | 0.962 |
| Female | 30 (30.0) | 122 (29.8) |  |
| Male | 70 (70.0) | 288 (70.2) |  |

CHD, coronary heart disease; SD, standard deviation.
