# Supplementary material for: Serum YKL-40 in coronary heart disease: linkage with inflammatory cytokines, artery stenosis, and optimal cut-off value for estimating major adverse cardiovascular events
Source: Front Cardiovasc Med. 2023 Oct 31;10:1242339. doi: 10.3389/fcvm.2023.1242339 (PMC10644235; doi:10.3389/fcvm.2023.1242339)
Supplement: Supplementary file 2 [file Table2.docx]

**Supplementary Table 2.** The correlation between YKL-40 and medication of CHD patients.

| Items | YKL-40 (ng/mL) | *Z* value | *P* value |
| --- | --- | --- | --- |
| β-blocker |  | -1.096 | 0.273 |
| No | 99.9 (71.3-139.9) |  |  |
| Yes | 100.8 (83.0-154.1) |  |  |
| Calcium channel blockers |  | -1.045 | 0.296 |
| No | 101.1 (82.6-153.6) |  |  |
| Yes | 99.3 (70.9-139.0) |  |  |
| Statin or other lipid-lowering therapy |  | -1.873 | 0.061 |
| No | 96.9 (75.5-144.9) |  |  |
| Yes | 104.5 (84.0-154.9) |  |  |
| ACEI or ARB |  | -1.323 | 0.186 |
| No | 98.7 (79.6-139.9) |  |  |
| Yes | 102.4 (78.9-162.5) |  |  |

YKL-40, chitinase-3-like protein 1; ACEI, angiotensin-converting enzyme inhibitor; ARB, angiotensin II receptor blocker.
