# Supplementary material for: Serum YKL-40 in coronary heart disease: linkage with inflammatory cytokines, artery stenosis, and optimal cut-off value for estimating major adverse cardiovascular events
Source: Front Cardiovasc Med. 2023 Oct 31;10:1242339. doi: 10.3389/fcvm.2023.1242339 (PMC10644235; doi:10.3389/fcvm.2023.1242339)
Supplement: Supplementary file 3 [file Table3.docx]

**Supplementary Table 3.** Univariate and stepwise forward multivariate Cox regression analysis of MACE.

| Factors | HR | 95% CI | | *P* value |
| --- | --- | --- | --- | --- |
|  |  | Lower | Upper |  |
| **Univariate** |  |  |  |  |
| YKL-40 ≥80 ng/mL, yes vs. no | 2.118 | 0.890 | 5.042 | 0.090 |
| YKL-40 ≥100 ng/mL, yes vs. no | 2.722 | 1.362 | 5.440 | 0.005 |
| YKL-40 ≥150 ng/mL, yes vs. no | 2.045 | 1.097 | 3.810 | 0.024 |
| Age ≥60 years, yes vs. no | 0.869 | 0.468 | 1.611 | 0.655 |
| Gender, male vs. female | 2.381 | 1.053 | 5.382 | 0.037 |
| BMI ≥28 kg/m^2^, yes vs. no | 0.838 | 0.386 | 1.819 | 0.654 |
| Smoke, former or current vs. never | 1.872 | 0.997 | 3.513 | 0.051 |
| Hypertension, yes vs. no | 1.266 | 0.620 | 2.584 | 0.517 |
| Hyperlipidemia, yes vs. no | 1.358 | 0.733 | 2.517 | 0.330 |
| DM, yes vs. no | 1.455 | 0.762 | 2.776 | 0.256 |
| CKD, yes vs. no | 0.913 | 0.405 | 2.061 | 0.827 |
| FBG ≥5.6 mmol/L, yes vs. no | 2.275 | 1.084 | 4.774 | 0.030 |
| Scr >110 μmol/L, yes vs. no | 1.502 | 0.628 | 3.594 | 0.361 |
| SUA >350 μmol/L, yes vs. no | 0.920 | 0.498 | 1.698 | 0.789 |
| TG ≥1.7 mmol/L, yes vs. no | 0.788 | 0.427 | 1.455 | 0.447 |
| TC ≥5.18 mmol/L, yes vs. no | 0.787 | 0.417 | 1.486 | 0.460 |
| LDL-C ≥2.60 mmol/L, yes vs. no | 0.467 | 0.216 | 1.012 | 0.054 |
| HDL-C ≥1.30 mmol/L, yes vs. no | 0.806 | 0.249 | 2.614 | 0.720 |
| CRP ≥5 mg/L, yes vs. no | 3.075 | 1.418 | 6.666 | 0.004 |
| Higher Gensini score, yes vs. no | 1.018 | 1.009 | 1.028 | <0.001 |
| Higher stenosis degree, yes vs. no | 1.776 | 1.215 | 2.596 | 0.003 |
| TNF-α ≥51.5 pg/mL, yes vs. no | 1.440 | 0.746 | 2.782 | 0.277 |
| IL-1β ≥1.0 pg/mL, yes vs. no | 0.864 | 0.453 | 1.650 | 0.659 |
| IL-6 ≥16.9 pg/mL, yes vs. no | 1.524 | 0.783 | 2.964 | 0.215 |
| IL-17A ≥58.6 pg/mL, yes vs. no | 1.045 | 0.548 | 1.992 | 0.894 |
| **Stepwise forward multivariate** |  |  |  |  |
| Smoke, former or current vs. never | 2.359 | 1.194 | 4.662 | 0.014 |
| SUA >350 μmol/L, yes vs. no | 0.505 | 0.256 | 0.997 | 0.049 |
| LDL-C ≥2.60 mmol/L, yes vs. no | 0.338 | 0.141 | 0.813 | 0.015 |
| CRP ≥5 mg/L, yes vs. no | 2.723 | 1.141 | 6.498 | 0.024 |
| Higher Gensini score, yes vs. no | 1.017 | 1.006 | 1.028 | 0.002 |

MACE, major adverse cardiovascular events; HR, hazard ratio; CI, confidence interval; YKL-40, chitinase-3-like protein 1; BMI, body mass index; DM, diabetes mellitus; CKD, chronic kidney disease; FBG, fasting plasma glucose; Scr, serum creatinine; SUA, serum uric acid; TG, triglyceride; TC, total cholesterol; LDL-C, low-density lipoprotein cholesterol; HDL-C, high-density lipoprotein cholesterol; CRP, C-reactive protein.
