# Supplementary figures and images for: Serum YKL-40 in coronary heart disease: linkage with inflammatory cytokines, artery stenosis, and optimal cut-off value for estimating major adverse cardiovascular events
Source: Front Cardiovasc Med. 2023 Oct 31;10:1242339. doi: 10.3389/fcvm.2023.1242339 (PMC10644235; doi:10.3389/fcvm.2023.1242339)

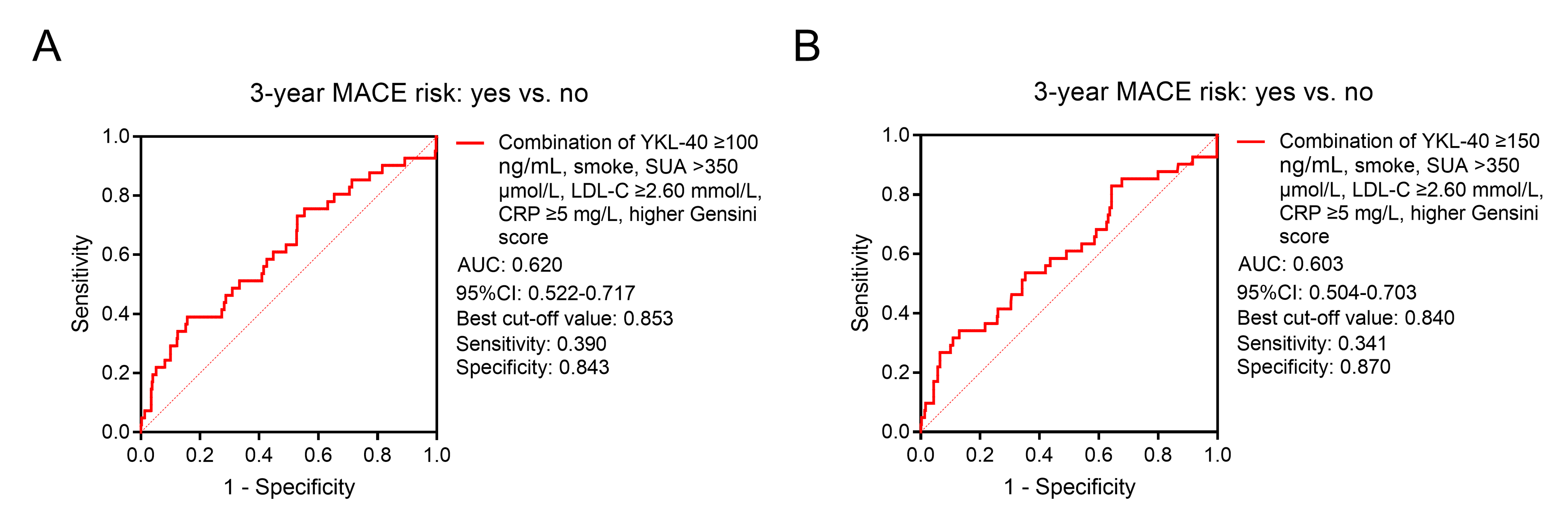

Supplement: Supplementary Figure S1 — Combination of serum YKL-40 and independent factors showed a general value for estimating 3-year MACE risks. The ROC curve for the ability of the combination of YKL-40 ≥100 ng/mL, smoke, SUA >350 μmol/L, LDL-C ≥2.60 mmol/L, CRP ≥5 mg/L, and higher Gensini score to predict 3-year MACE risk in CHD patients (A). The ROC curve for the ability of the combination of YKL-40 ≥150 ng/mL, smoke, SUA >350 μmol/L, LDL-C ≥2.60 mmol/L, CRP ≥5 mg/L, and higher Gensini score to predict 3-year MACE risk in CHD patients (B). Fix correct style. [file Image1.tif]
